# Modelling the Species Distribution of Flat-Headed Cats (*Prionailurus planiceps*), an Endangered South-East Asian Small Felid

**DOI:** 10.1371/journal.pone.0009612

**Published:** 2010-03-17

**Authors:** Andreas Wilting, Anna Cord, Andrew J. Hearn, Deike Hesse, Azlan Mohamed, Carl Traeholdt, Susan M. Cheyne, Sunarto Sunarto, Mohd-Azlan Jayasilan, Joanna Ross, Aurélie C. Shapiro, Anthony Sebastian, Stefan Dech, Christine Breitenmoser, Jim Sanderson, J. W. Duckworth, Heribert Hofer

**Affiliations:** 1 Leibniz Institute for Zoo and Wildlife Research, Berlin, Germany; 2 Department of Remote Sensing, Institute of Geography, University of Würzburg, Würzburg, Germany; 3 German Remote Sensing Data Center (DFD), German Aerospace Center (DLR), Wessling, Germany; 4 Global Canopy Programme, Oxford, United Kingdom; 5 Wildlife Conservation Research Unit, University of Oxford, Oxford, United Kingdom; 6 German Institute of Human Nutrition, Department of Experimental Diabetes, Potsdam-Rehbrücke, Germany; 7 Institute for Tropical Biology and Conservation, University Malaysia Sabah, Kota Kinabalu, Malaysia; 8 WWF-Malaysia, Selangor, Malaysia; 9 Copenhagen Zoo, Department of Research and Conservation, Frederiksberg, Denmark; 10 Orang-utan Tropical Peatland Project, University of Palangka Raya, Kalimantan, Indonesia; 11 Department of Fisheries and Wildlife Sciences, Virginia Tech, Blacksburg, Virginia, United States of America; 12 WWF-Indonesia, Pekanbaru, Indonesia; 13 Department of Zoology, Faculty of Resource Science and Technology, Kota Samarahan, Sarawak, Malaysia; 14 School of Environmental Research, Charles Darwin University, Northern Territory, Australia; 15 Conservation Science Program, WWF-US, Washington, District of Columbia, United States of America; 16 Aonyx Environmental, Kuching, Sarawak, Malaysia; 17 IUCN/SSC Cat Specialist Group, c/o KORA, Muri b. Bern, Switzerland; 18 Small Wild Cat Conservation Foundation, Wildlife Conservation Network, Los Altos, California, United States of America; 19 Wildlife Conservation Society Asia Program, New York, New York, United States of America; University of Pretoria, South Africa

## Abstract

**Background:**

The flat-headed cat (*Prionailurus planiceps*) is one of the world's least known, highly threatened felids with a distribution restricted to tropical lowland rainforests in Peninsular Thailand/Malaysia, Borneo and Sumatra. Throughout its geographic range large-scale anthropogenic transformation processes, including the pollution of fresh-water river systems and landscape fragmentation, raise concerns regarding its conservation status. Despite an increasing number of camera-trapping field surveys for carnivores in South-East Asia during the past two decades, few of these studies recorded the flat-headed cat.

**Methodology/Principal Findings:**

In this study, we designed a predictive species distribution model using the Maximum Entropy (MaxEnt) algorithm to reassess the potential current distribution and conservation status of the flat-headed cat. Eighty-eight independent species occurrence records were gathered from field surveys, literature records, and museum collections. These current and historical records were analysed in relation to bioclimatic variables (WorldClim), altitude (SRTM) and minimum distance to larger water resources (Digital Chart of the World). Distance to water was identified as the key predictor for the occurrence of flat-headed cats (>50% explanation). In addition, we used different land cover maps (GLC2000, GlobCover and SarVision LLC for Borneo), information on protected areas and regional human population density data to extract suitable habitats from the potential distribution predicted by the MaxEnt model. Between 54% and 68% of suitable habitat has already been converted to unsuitable land cover types (e.g. croplands, plantations), and only between 10% and 20% of suitable land cover is categorised as fully protected according to the IUCN criteria. The remaining habitats are highly fragmented and only a few larger forest patches remain.

**Conclusion/Significance:**

Based on our findings, we recommend that future conservation efforts for the flat-headed cat should focus on the identified remaining key localities and be implemented through a continuous dialogue between local stakeholders, conservationists and scientists to ensure its long-term survival. The flat-headed cat can serve as a flagship species for the protection of several other endangered species associated with the threatened tropical lowland forests and surface fresh-water sources in this region.

## Introduction

**Figure 1:**
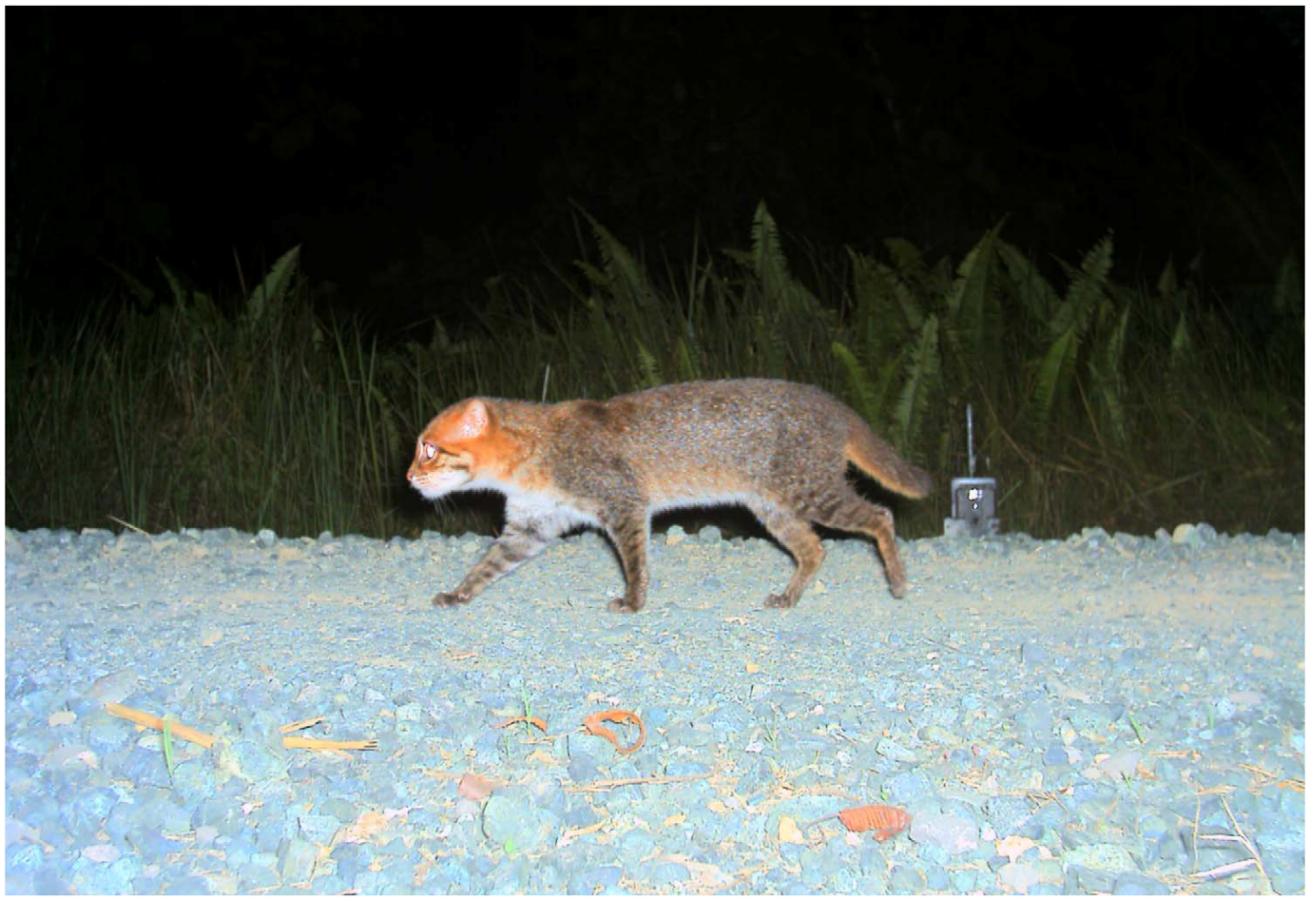
Photo of a flat-headed cat, camera-trapped in Tangkulap Forest Reserve, Sabah, Malaysia in March 2009.

**Figure 2:**
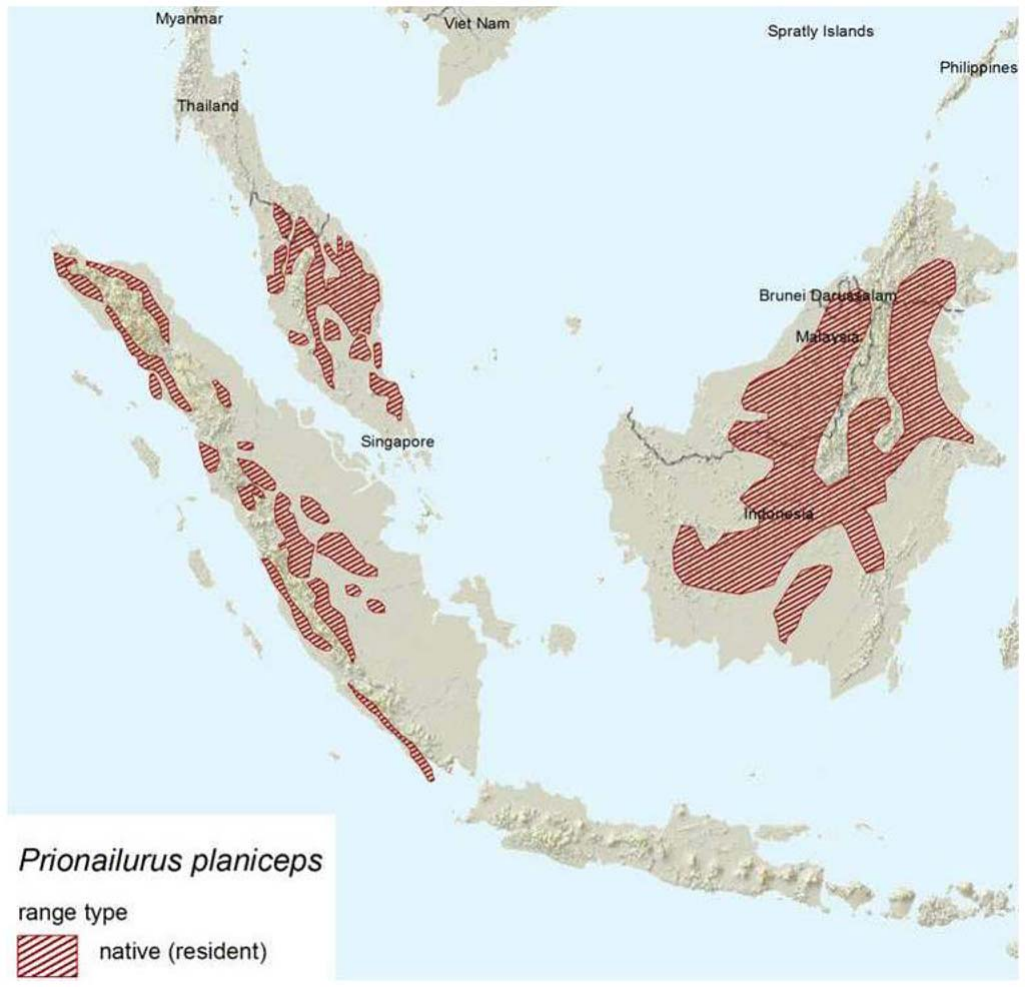
Geographical range of the flat-headed cat according to the IUCN red list 2008 (http://www.iucnredlist.org/details/18148/0/rangemap).

The diminutive flat-headed cat *Prionailurus planiceps* (Vigors and Horsfield, 1827), with adults weighing as little as 1.59 kg [1] (Figure 1), occurs in southern Thailand, Peninsular Malaysia, Sumatra and Borneo [2]-[4] (Figure 2). Some authors also suggest that its range extends into the southern part of Myanmar [5]. This species has never been studied intensively in the wild [1], [5]–[6]. In 2008, a revision of the IUCN Red List suggested an increasing risk of extinction for the flat-headed cat and its status was changed from “Vulnerable” to “Endangered” [3].

Most information on the distribution and natural history of the flat-headed cat comes from opportunistic incidental sightings, specimen collectors [1], [5]–[6], and a few animals kept in captivity [7]–[8]. Morphological adaptations such as its dental structure [9]–[10] and the slight web between the toes as well as incidental observations suggest that this cat is well adapted to hunt small prey in shallow water and on muddy shores [7]. For instance, Leyhausen reported that a captive flat-headed cat displayed more interest in a mouse in a pond than on land [7], and the stomach of an adult shot at a riverbank contained only fish [1]. Individuals have been observed or collected in undisturbed primary and secondary forests mostly along rivers, streams and in flooded areas [1], [6], [11]. Only Khan [in 5] has reported sightings in oil palm plantations and even speculated that this species benefits from the expansion of these plantations in Peninsular Malaysia [12]. However, as we are aware of no other similar records from anthropogenically transformed habitats, it is unlikely that flat-headed cats survive and reproduce in palm oil or rubber plantations. Nothing is known regarding the home-range sizes and population densities of flat-headed cats and their presumably nocturnal behaviour (considering the records obtained in this study) makes surveying and monitoring difficult. Although camera-trapping efforts have increased in magnitude and extent during the past few years [13], only a few studies have recorded this species, with the number of photographs being so low that abundance estimates could not be calculated.

Several developments in ecological niche modelling (ENM) have provided new tools to estimate species ranges and identify suitable habitats [14]–[21]. For many threatened species, only a few historical (museum) and recent records are available which may result in a low accuracy and a poor fit of most ecological niche models [17], [22]. However, the maximum entropy (MaxEnt) framework [23] appears to be robust even if only few occurrence records are available [24]–[26]. Although ENM has been used to predict the distribution of little-known carnivores [15], [17], [20], we are not aware of any application of this framework to assess the potential distribution of any Asian felid species.

The aim of this study is to describe the historical and current distribution of the flat-headed cat, predict its potential habitat occupancy by applying a MaxEnt model throughout its range, identify key localities suitable to contribute to the long-term survival of the species, and ultimately contribute to an update of the conservation status of this endangered species.

## Materials and Methods

### Species Occurrence Samples

In January 2009, during the Clouded Leopard and Small Cat Summit organised by the IUCN Cat Specialist Group and the IUCN Conservation Breeding Specialist Group, only 14 records of the flat-headed cat were compiled by the attending scientists and conservationists [27]. We collected additional records from the existing scientific literature and researchers who were unable to join the summit and conducted a survey in several natural history museums. We only included reports of direct sightings, camera-trapping pictures or dead specimens. We considered all reported locations to be accurate for species identification since the flat-headed cat has a very distinctive appearance and is not easily mistaken for any other felid or other small carnivore. Owing to their unknown origin, wild caught zoo animals kept in Thailand and Malaysia were not included in the analysis. We also included the results of a questionnaire conducted by MAJ in Sarawak (northwest Borneo) as the interviewees were familiar with the flat-headed cat.

A total of 107 records (47 historical and 60 recent) was obtained (Table S1). We checked for and excluded all multiple occurrences of sites (particularly within museum records), resulting in a final number of 88 independent records (29 historical and 59 recent) with a minimum distance of one km between the records. Records were defined as recent if they were collected within the last 25 years (since 1984); the majority was obtained during the last 10 years, although the precise year was not available in all cases. The oldest historical record from the *Museum für Naturkunde* in Berlin dated back to 1864. For 21 recent records precise geographic coordinates were available, for the remainder a general description of the locality. For these records we assigned coordinates using published resources such as Google Earth 5.0.1 or the database of BirdLife International (www.birdlife.org). We assume that the positional accuracy of the geographic coordinates assigned to the records was between one and ten kilometres.

### MaxEnt Model

A series of bioclimatic variables including eleven temperature and eight precipitation metrics was obtained from the WorldClim database (Version 1.4, http://www.worldclim.org/bioclim.htm) [28]. WorldClim data are derived from monthly temperature and precipitation values using long-term time series from 1950 to 2000 from a global network of 4,000 climate stations with a spatial resolution of one km^2^. The WorldClim parameters express spatial variation in annual means, seasonality and extreme or limiting climatic factors and represent biologically meaningful variables for characterising species distributions [29]. Topographic data acquired during the SRTM mission and re-sampled from a 90×90 m^2^ to a 1×1 km^2^ spatial resolution were downloaded from the WorldClim data base. Surface water bodies (rivers and lakes) were extracted from the country inland water data of the Digital Chart of the World (DCW; http://biogeo.berkeley.edu/bgm/gdata.php) and the minimum distance to water in km was calculated per pixel using ArcGIS 9.3 software and used as an input variable for the model. These abiotic variables were then analysed together with the species occurrence data using the Maximum Entropy algorithm as implemented in MaxEnt software Version 3.2.1. Ten individual MaxEnt models were run in batch mode with the following settings: Auto features (feature types are automatically selected depending on the training sample size), perform jackknife tests, logistic output format, random test percentage  = 25, regularisation multiplier  = 1, maximum iterations  = 1000, convergence threshold  = 0.0001 and maximum number of background points  = 10,000. We used the mean probabilities predicted by the ten independent models as estimates for subsequent analyses. The MaxEnt model prediction was regarded as the potential former distribution, since historical and recent occurrences were treated with equal weight in the models.

As the modelling algorithm is a major source of uncertainty in the prediction of species distribution [30]–[32] we assessed the robustness of the results of the MaxEnt algorithm by comparing them with the results of two other algorithms, Environmental Distance and Support Vector Machines (SVM) as implemented in the openModeller software (http://openmodeller.sourceforge.net). We computed the area under the receiver operating characteristic curve (AUC) to assess model discriminatory power for the different algorithms. We further compared the predicted probabilities of occurrence for 5,000 points randomly distributed throughout the land surface within the study region between the MaxEnt, SVM and Environmental Distance models using Pearson's correlation coefficient *r* [33].

To extract suitable habitats from the predicted continuous surfaces of mean probabilities of occurrence we created five different scenarios using 0%, 10%, 20%, 30% and 40% omission thresholds (including both training and test samples). Subsequent analyses were done for all scenarios, but the maps shown were based on a conservative approach (10% omission threshold).

### Land Cover within Predicted Distribution

As we included both historical and recent records in the analysis, the inclusion of land cover information in the model was not feasible owing to the time elapsed between historical records and the collection of land cover data, and the extent of habitat modification during this period. We therefore used land cover information to refine the predicted former distribution of the flat-headed cat. Three land cover maps were chosen for this purpose: the Global Land Cover 2000 (GLC 2000), the GlobCover Land Cover (GlobCover) Version 2.1 and–available only for Borneo - a classification developed by SarVision LLC (Table 1).

**Table 1:** Land cover maps used in this study.

Product	Short name	Year	Spatial resolution	Sensor	Produced by	Download
Global Land Cover 2000	GLC 2000	2000	1000 m	SPOT-4	Global Vegetation Monitoring Unit (European Commission)	http://bioval.jrc.ec.europa.eu/products/glc2000/products.php
GlobCover (Version 2.1)	GlobCover	2005 - 06	300 m	ENVISAT- MERIS	GlobCover Project	http://ionia1.esrin.esa.int/
SarVision LLC*	SarVision	2007	232 m	MODIS	SarVision LLC	-

*This land cover map was only available for Borneo.

Based on our field records and the limited existing knowledge about appropriate habitats for the flat-headed cat, we reclassified all land cover maps into five categories (Table 2): 0 =  unsuitable habitat (croplands, bare or burnt areas, artificial areas, upper montane forest), 1 =  very poor habitat (mosaic cropland/vegetation, lower montane forest, closed to open evergreen shrubland), 2 =  poor habitat, maybe suitable as a corridor (mosaic vegetation, upland forests), 3 =  good habitat (lowland forest) and 4 =  very good habitat (regularly or permanently flooded forest, peat swamp forest, mangroves).

**Table 2:** Land cover reclassification scheme used for habitat suitability analysis.

Dataset		Original class	Reclassified class
**GlobCover**			
Irrigated–croplands; - shrub or tree crops		11; 12	0
Rainfed–croplands		14	0
Mosaic–Croplands (50–70%)/Vegetation (20–50%)		20	1
Mosaic–Vegetation (50–70%)/Croplands (20–50%)		30	2
Closed to open broadleaved evergreen or semi-deciduous forest		40	3
Closed needleleaved evergreen forest		70	1
Mosaic Forest-Shrubland (50–70%)/Grassland (20–50%)		110	2
Closed to open shrubland		130	1
Closed to open grassland		140	0
Closed to open broadleaved forest regularly flooded (fresh-brackish water)		160	4
Closed broadleaved forest permanently flooded (saline-brackish water)		170	4
Artificial areas		190	0
**GLC 2000**			
Tree cover, broadleaved, evergreen		1	3
Tree cover, regularly flooded, fresh water		7	4
Tree cover, regularly flooded, saline water		8	4
Mosaic tree cover/other natural vegetation		9	2
Shrub cover, closed–open, evergreen		11	1
Sparse herbaceous or sparse shrub cover		14	0
Cultivated and managed areas		16	0
Mosaic cropland/tree cover/other natural vegetation		17	0
Mosaic cropland/shrub and/or other natural vegetation		18	0
Bare areas		19	0
Water bodies		20	0
No data		22	0
**SarVision LLC** *			
Lowland forest		1	3
Upland forest		2	2
Lower montane forest		3	1
Upper montane forest		4	0
Swamp forest		5	4
Mangrove		6	4
Old plantations		7	0
Yung plantations and crops		8	0
Burnt forest area		9	0
Mixed crops		10	0
Bare area		11	0
Water and fishponds		12	0
Water		13	0
No data		14	0

*This land cover map was only available for Borneo.

We then analysed the current land cover status at both historically and recently recorded localities. For this purpose, we created a buffer zone around each locality representative for the ecological requirements of the flat-headed cat. Because actual home range sizes of flat-headed cats are unknown, we used data on home-range sizes of the leopard cat *Prionailurus bengalensis* as an approximation, a closely related, similar sized, sympatric felid. Known home-range sizes for female leopard cats vary between 1.75 km^2^ on Iriomote Island [34] and 2.1 km^2^ on Borneo [35], and for males between 5.8 km^2^ [36] and 7.5 km^2^ [37] in Thailand. We took the rounded mean value of these extremes of 4 km^2^ as an estimate of the home-range size of the flat-headed cat. To accommodate uncertainties associated with the precise position of point localities we used a buffer area of three times the extrapolated home-range (12 km^2^). This analysis was conducted using the Spatial Analyst extension of ArcGIS 9.3 for all three land cover classifications. If reclassification of land cover habitats was appropriate, then the reclassified habitat types in the buffer areas surrounding recent records should be mainly in categories 3 and 4. We also assessed the habitats in the buffer areas around historical records to detect whether they have already been transformed into unsuitable environmental conditions.

### Human Population Density

To refine initial model predictions, human population density data supplied by the LandScan 2007™ High Resolution Global Population Data Set (UT-Battelle, LLC) compiled on a 30″×30″ latitude/longitude grid were used. We reclassified human population densities into 5 categories: class 0 =  more than 25 inhabitants km^−^^2^ (unsuitable for the occurrence of flat-headed cats), class 1 = 10–25 inhabitants km^−^^2^, class 2 = 5–10 inhabitants km^−^^2^, class 3 = 1–5 inhabitants km^−^^2^, class 4 = 0 inhabitants km^−^^2^ (no human disturbance, presumably optimal for flat-headed cats). As with the land cover data, we extracted human population densities in buffer areas of 12 km^2^ around historical and recent records.

### Estimating the Loss of Suitable Habitats

The major threat for flat-headed cats is presumably the transformation of their habitats to arable land or plantations [e. g. 3]. We therefore assessed the loss of suitable habitats owing to land cover conversion for the five model scenarios. The proportions of the reclassified land cover classes were extracted and classes 3 and 4 defined as appropriate habitats for the flat-headed cat. The relative proportion of these two classes in relation to the area with non-suitable cover types (classes 0–2) was taken as a measure of the loss of suitable habitat caused by anthropogenic factors.

### Key Localities and Protected Areas

To identify habitats with a high probability of current occurrence of flat-headed cats we calculated a habitat suitability index (HSI) [38]. For this purpose, we multiplied the averaged prediction of the ten MaxEnt model runs with the categorised land cover classes and with the reclassified human population density classes. As we assume that the land cover and the predictions by the MaxEnt model are more important than the human population density for the occurrence of flat-headed cats we doubled the weight of these variables. To calculate the HSI of flat-headed cats in each pixel (resolution 1 km^2^) we modified the equation of Allen *et al*. [38] to read HSI  = (M^2^×L^2^×H)^1/5^, where M is the mean probability predicted by the ten MaxEnt models, L is the land cover class and H is the human population density category. In order to scale the HSI values between 0 and 1, we rescaled the land cover classes and human population density classes as follows: class 4 was rescaled to 1, class 3 to 0.75, class 2 to 0.5, class 1 to 0.25 and class 0 kept its value. Thus, if any component is unsuitable ( =  0) the HSI will be 0 as well. The application of this equation ensures that even an area with a relatively high human population density (class 1) would retain a high probability to harbour flat-headed cats if both the mean probability predicted by the MaxEnt model and its land cover class are favourable (class 3 or 4). Based on the HSI maps we could identify large contiguous forest blocks with potentially suitable habitat for the flat-headed cat.

The 2009 World Database of Protected Areas (www.wdpa.org) was used to determine the level of legal protection throughout the study area and the proportion of potential habitat within totally protected areas. For this aim, potential habitats were defined as areas covered with vegetation and human population classes 3 or 4 within the predicted potential former distribution for all five threshold scenarios. For comparison we analysed the proportion of all forested areas classified as totally protected under the IUCN criteria in the study region.

### Statistical Analysis

Where appropriate, results are presented as means ± standard deviation. We used chi-square distributed log-likelihood G tests as implemented in Systat (version 12, Systat Inc., San Jose, CA, USA) to test for differences between buffer areas of historical and recent records in the distribution of forest classes and human population densities. If the values for land cover or human population density categories for each pixel within a buffer zone are spatially autocorrelated, then neighbouring pixel values would carry less information than truly independent values and the calculated p-value for the log-likelihood G will underestimate the true type I error probability [39]. The appropriate strategy is to estimate the “effective sample size” by accounting for possible spatial autocorrelation and adjusting the log-likelihood G value accordingly [40]. Because we were not aware of a published approach to correct for spatial autocorrelation in 2 x k contingency tables, we proceeded as follows: We followed Cerioli [41] who, for 2×2 contingency tables, divided the log-likelihood G by a correction factor (1+λ) where λ is a measure of the degree of spatial autocorrelation for the relevant lattice (e.g., an appropriate summation of Moran's spatial autocorrelation coefficient computed over an entire contiguous lattice). The buffer zones for our records are disjointed rather than contiguous, and therefore, spatial autocorrelation between buffer zones is unlikely and Cerioli's λ, computed over the entire distribution map, would be unduly conservative. We therefore assumed that there was no spatial autocorrelation between buffer zones. Instead, to protect type I error probabilities we assumed that significant spatial autocorrelation did occur across the entire buffer zone around each flat-headed cat record and calculated an indicator λ for it as (i) the maximum order number of neighbouring pixels in relation to the central pixel of each buffer zone  =  ((√number of pixels per buffer zone)-1)/2; (ii) the maximum number of distance classes between pixels of a buffer zone  =  (√number of pixels per buffer zone)-1. The log-likelihood G would then, in analogy to [40], be adjusted by dividing the original G by the correction factor  =  (1+λ) and calculating the type I error probability for the adjusted G using the chi-square distribution and the same degrees of freedom as for the original G. These adjustments reduced the log-likelihood G but did not substantially affect the p-values (Table 3). With the most extreme approach, i.e., treating all buffer zone pixels as if they were identical and using the number of pixels per buffer zone as correction factor, the p–values for the comparison of historical and recent records were still significant for the GLC2000 (p = 0.044) and SarVision LLC maps (p = 0.00027) in terms of habitat suitability and for human population density (p = 0.0023), only the GlobCover map produces then a p of 0.16.

**Table 3:** Log-likelihood ratio tests on differences between buffer zones around recent and historical records when reclassified in terms of habitat suitability for flat-headed cats (see Table 2) or human population density (see Materials and Methods).

	Log-likelihood G	Average number of data points per buffer zone (pixels)	λ (value for indicator of possible spatial autocorrelation)	Correction factor (1+λ) †	Adjusted log-likelihood G ‡	df	Adjusted type I error probability (p-value)						
Indicator of possible spatial autocorrelation: average order number of pixels in relation to the central pixel for each buffer zone													
GLC2000 habitat suitability	113.347	11.540	1.199	2.199	51.556	4	<0.00001						
GlobCover habitat suitability	663.361	101.140	4.528	5.528	119.991	4	<0.00001						
SarVision LLC* habitat suitability	2674.893	125.085	5.092	6.092	439.078	4	<0.00001						
Human population density	193.097	11.655	1.207	2.206	87.494	4	<0.00001						
Indicator of possible spatial autocorrelation: maximum distance class within a buffer zone													
GLC2000 habitat suitability	113.347	11.540	2.397	3.397	33.366	4	<0.00001						
GlobCover habitat suitability	663.361	101.140	9.057	10.057	65.961	4	<0.00001						
SarVision LLC* habitat suitability	2674.893	125.085	10.184	11.184	239.168	4	<0.00001						
Human population density	193.097	11.655	2.414	3.414	56.561	4	<0.00001						

†correction factor to adjust log-likelihood ratio G for possible spatial autocorrelation.

‡corrected for possible spatial autocorrelation.

*This land cover map was only available for Borneo.

## Results

### Flat-Headed Cat Records

**Figure 3:**
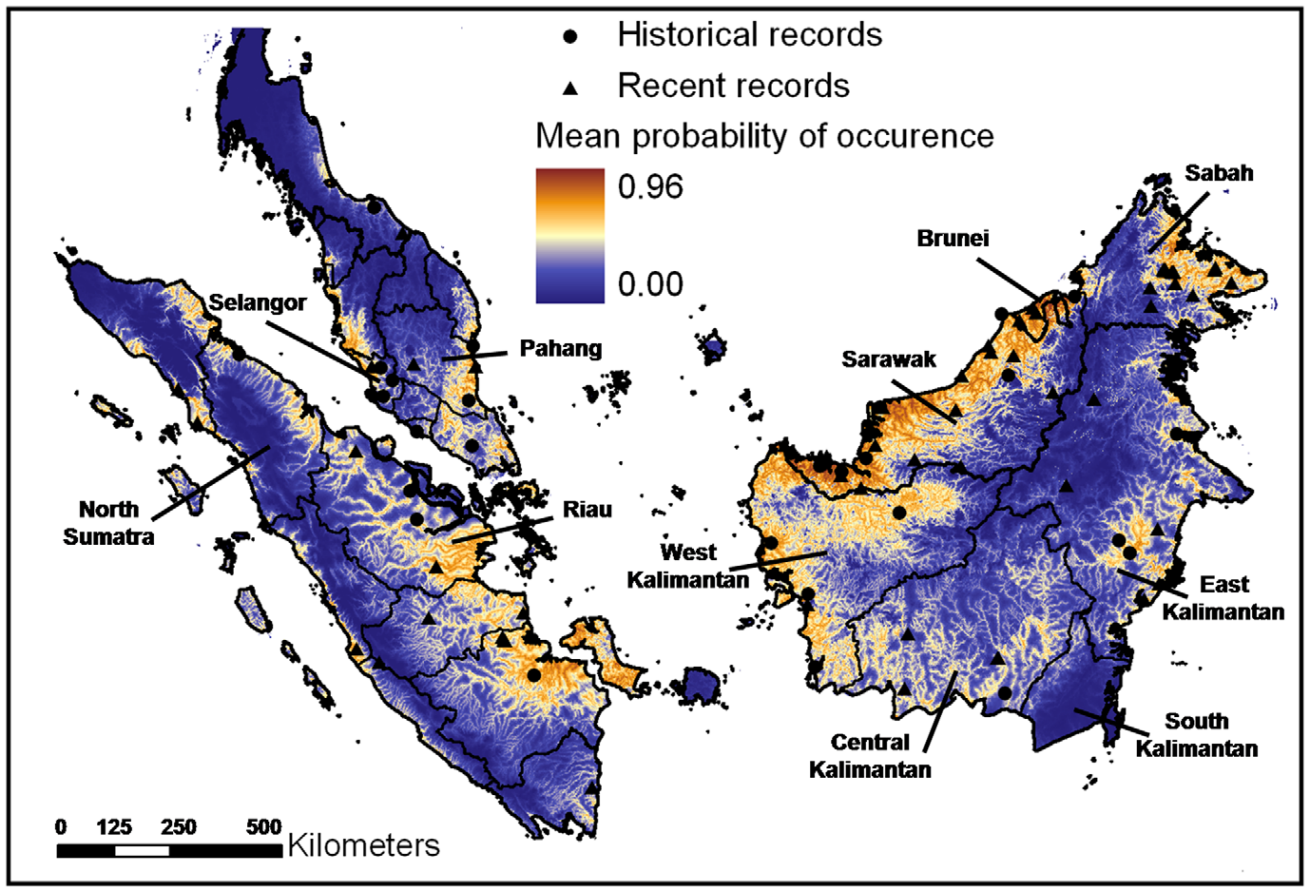
Predicted former distribution of the flat-headed cat according to the mean MaxEnt model including 21 topo-climatic variables. Circles indicate the location of historical, triangles the location of recent records.

Our literature survey and contact with scientists currently working in the field yielded several new records of the flat-headed cat from Thailand, Malaysia, Brunei Darussalam and Indonesia. Table S1 contains a complete list of all recent and historical records we had access to. Most recent records (43) came from Borneo: three were recorded in Brunei Darussalam, 12 in Kalimantan, Indonesian Borneo and 28 in Malaysian Borneo (14 in Sabah and 14 in Sarawak). We also obtained information on the origin of 27 museum specimens (17 geographically independent) collected in Borneo in historical times. From Sumatra we obtained 13 independent recent and only four independent historical records, since most of the museum specimens did not have a precise collection location. From the southern part of Thailand and from Peninsular Malaysia we received recent records from four forest patches (Toh Daeng Peat Swamp forest in Thailand, Selangor Peat Swamp Forest, Krau Game Reserve and Pahang Peat Swamp Forest in Malaysia) and 14 historical records (8 were geographically independent). None of the scientists working in the southern part of Myanmar could confirm the presence of the flat-headed cat there (Su Su *in litt.*, Anthony Lynam *in litt*., Nay Myo Shwe *in litt.*). Figure 3 depicts localities of recent and historical records.

For 88 out of a total of 107 records, geographic coordinates had to be estimated from descriptions of localities. Photographs from camera-traps or direct sightings of flat-headed cats with precise location information were available for 19 locations (15 on Borneo and four on Sumatra). During one of these sightings along a small tributary of the Kinabatangan River in Sabah (Malaysia) two authors (AW & AM) filmed a wild flat-headed cat for several minutes (Video S1).

### MaxEnt Models and Their Robustness

For all ten models, the fit as measured by the mean area under the curve (AUC) of the receiver operating characteristic (ROC) was high, with mean values of 0.982 (0.980 to 0.983) for the training data and 0.968 (0.966 to 0.977) for the test data (Table 4). Similarly, the model's discriminatory power (measured by AUC) of the two other modelling algorithms yielded values of 0.89 for Support Vector Machines (SVM) and 0.99 for Environmental distance. The comparison of the models demonstrated strong similarity of the predicted distributional areas between MaxEnt and the other modelling algorithms (correlation between MaxEnt and Environmental Distance *r* = 0.752, N = 5,000, p<0.01; between MaxEnt and SVM *r* = 0.831, N = 5,000, p<0.01). Based on this spatial agreements between the different model algorithms we consider the results and conclusions from the MaxEnt model to be robust. Therefore, we present and discuss only the results of the MaxEnt model.

**Table 4:** Area under curve (AUC) values of the receiver operating characteristic (ROC) for the ten MaxEnt models.

MaxEnt model	1	2	3	4	5	6	7	8	9	10
Training data	0.982	0.980	0.983	0.983	0.980	0.983	0.982	0.982	0.98	0.983
Test data	0.971	0.976	0.959	0.961	0.971	0.966	0.971	0.970	0.977	0.969

The most important abiotic variable was *minimum distance to water*, explaining 53.6±2.9% of variation in the distribution of the flat-headed cat. The omission of *minimum distance to water* as the main explanatory variable significantly decreased the gain of the model, indicating its important information content not present in the other 20 input variables. The second most important variable was *precipitation of the driest month*, explaining 13.6±8.7% of the variance. The third most important variable was *altitude* explaining 8.0±2.2% of the model variance. Figure 3 compares localities of historical and recent records with the distribution of the flat-headed cat as predicted by the mean MaxEnt model. Areas with high probabilities of predicted occurrence were mainly confined to lowland areas.

### Environmental Conditions at Sites of Records

**Figure 4:**
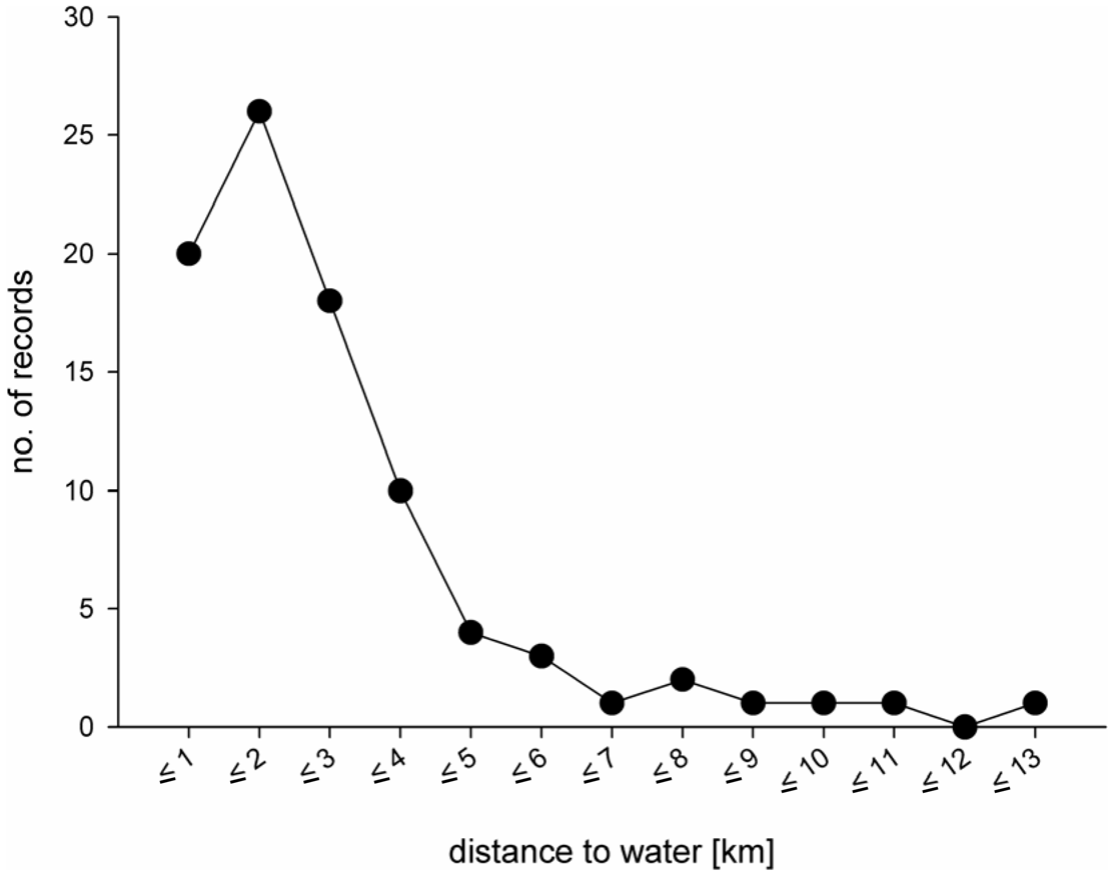
Distance of flat-headed cat records to major water resources (lakes and rivers).

**Figure 5:**
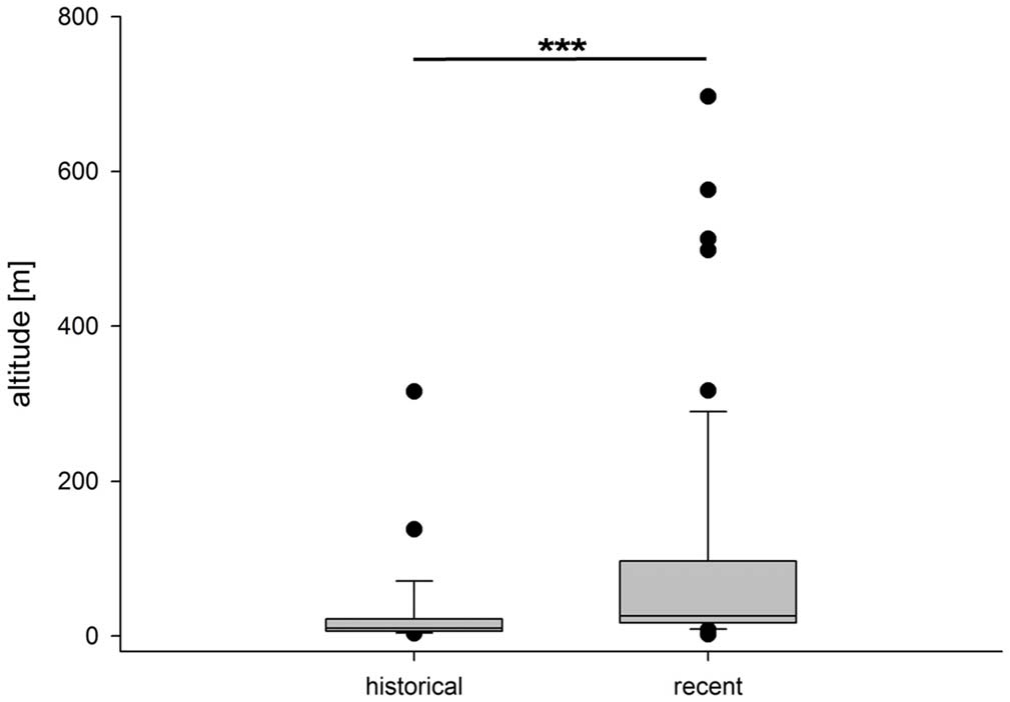
Box plot diagram representing median (mid-line), interquartile range (shaded boxes), range (whiskers) and outliers (dots) of the altitude for historical and recent flat-headed cat records (Mann-Whitney U-test, U = 402, N = 88, p<0.001).

Our results are consistent with the assumption that the occurrence of flat-headed cats primarily depends on the availability of freshwater habitats. Over 70% of records were collected less than three kilometres away and only four records (<5%) were located more than eight kilometres away from the nearest major river or lake (Figure 4). As improved access to remote areas today may have extended sampling to areas historically little sampled, we tested for possible sampling biases in both historical and recent records. There was no difference between the distributions of distances to freshwater habitats of historical and recent records (Mann-Whitney U-test, U = 744, N = 88, p = 0.31). In contrast, recent records were significantly more likely to be located at higher altitudes than historical records (U = 402, N = 88, p<0.001), although almost 80% of recent and more than 90% of historical records are located below 100 m asl (Figure 5).

### Land Cover within the Predicted Distribution and Loss of Suitable Habitat

**Figure 6:**
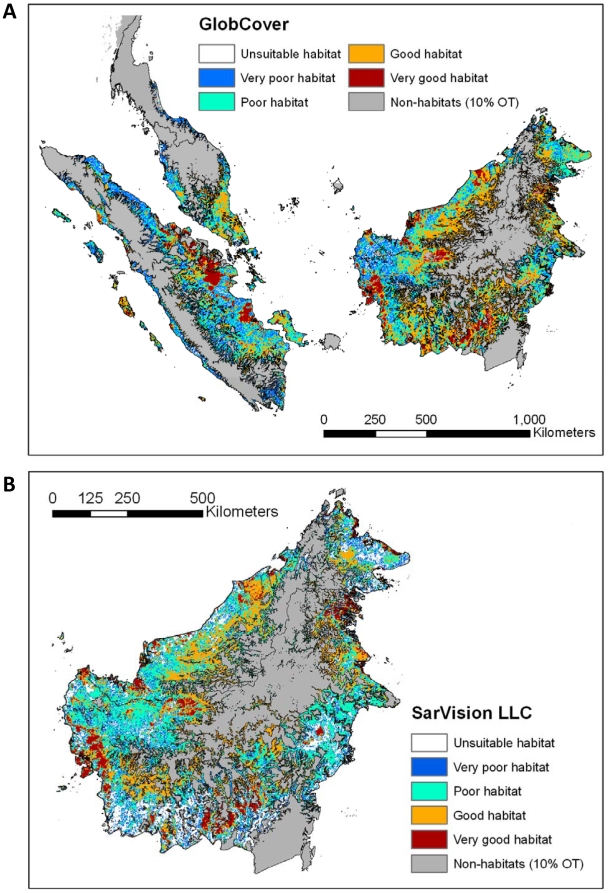
Land cover categories (GlobCover, SarVision LLC) within the modeled distribution range of the flat-headed cat. Results are shown for a strict scenario allowing only 10% omission of all samples.

**Figure 7:**
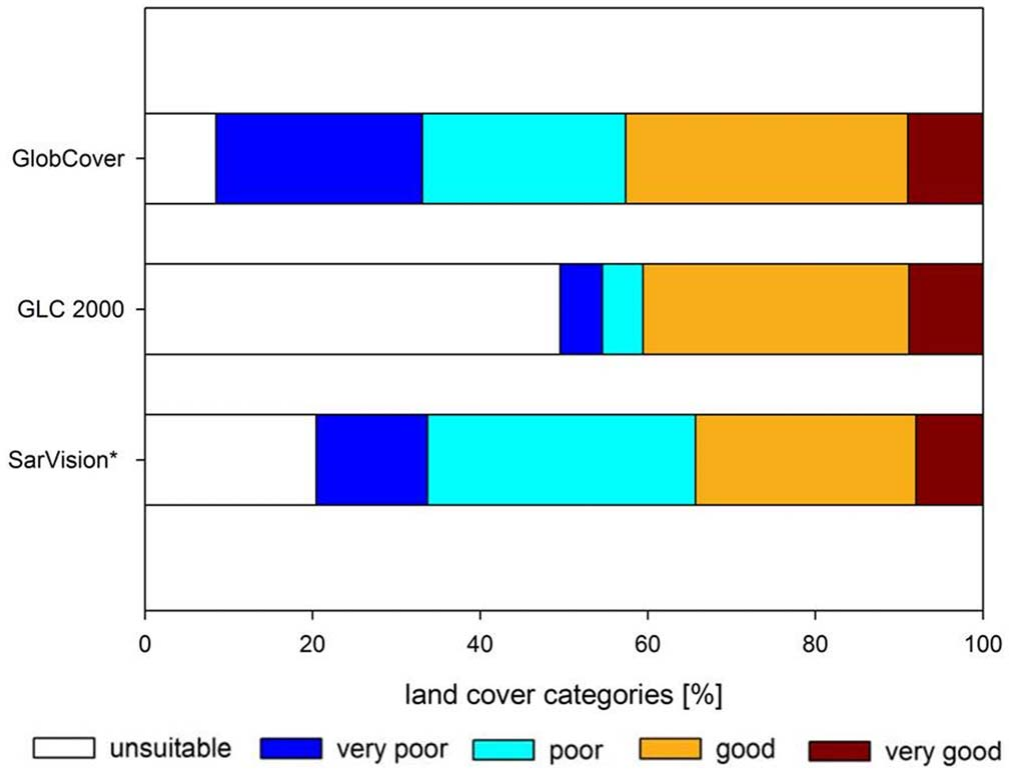
Land cover categories within the proportion of the predicted former distribution of the flat-headed cat as captured by the 10% omission threshold scenario.

Figure 6a shows the reclassified GlobCover land cover map for areas predicted to be suitable habitats for the flat-headed cat when applying a 10% omission threshold. Figure 6b illustrates the same results projected onto the SarVision data set of Borneo, the most precise vegetation classification and the only one which distinguishes between montane, upland and lowland forest. Many of the predicted potentially suitable historical habitats were assigned to the unsuitable or poor classes 0 to 2 in all three land cover maps. The relative proportion of already degraded habitat (e.g. arable land, plantations, mosaic vegetation) was similar for all omission threshold scenarios (0%, 10%, 20%, 30% and 40% thresholds) between the three land cover maps (Table 5). The total estimate of loss of suitable habitat varied between 54% (GlobCover 0% omission threshold) and 68% (SarVision 0% omission threshold) (Figure 7). As the SarVision land cover classification only covered Borneo, comparisons of relative losses between different land cover maps may be of limited use.

**Table 5:** Proportion of unsuitable or poor land cover (classes 0–2) within predicted areas of occurrence.

	0%	10%	20%	30%	40%
GlobCover	0.5355	0.5737	0.5906	0.5972	0.6061
GLC 2000	0.5451	0.5943	0.6074	0.6033	0.6004
SarVision*	0.6845	0.6571	0.6682	0.6691	0.6717

Results are shown for the three different land cover classifications and five omission threshold scenarios and provide an indication of the loss of suitable habitat through anthropogenic habitat modification. * This land cover map was only available for Borneo.

**Figure 8:**
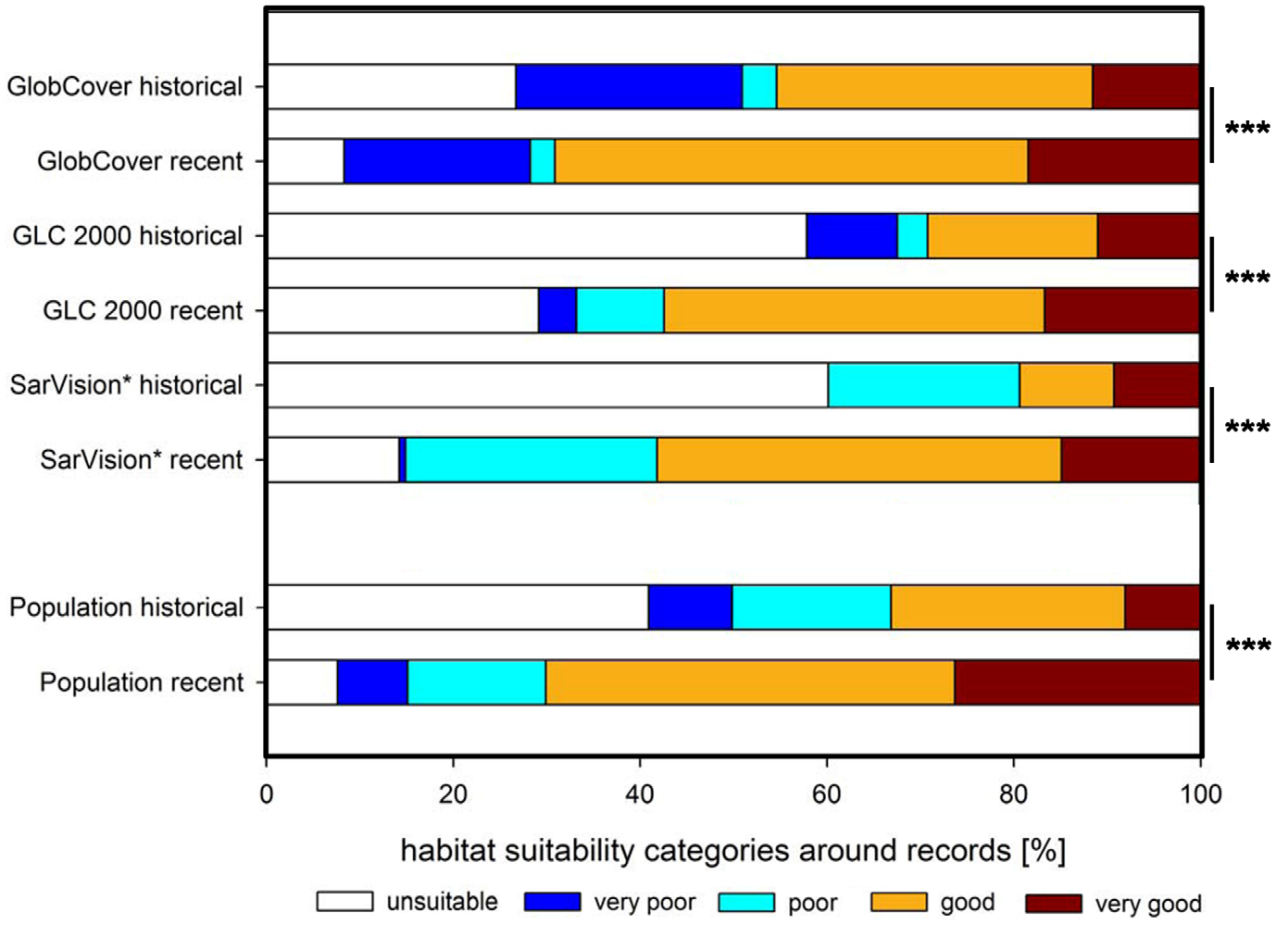
Proportions of habitat suitability categories (land cover and human population density classes) within 12 km^2^ buffers around historical and recent flat-headed cat records (log-likelihood G test p<0.001).

The landscape around historical records is significantly more degraded than around recent records (Table 3). In the buffer zones around historical records most suitable habitat has already been modified into unsuitable or poor land cover (classes 0–2) (54% GlobCover, 72% GLC 2000 and 80% SarVision). Between 31% (GlobCover) and 42% (GLC 2000 and SarVision) of buffer areas around recent records were categorised in land cover classes 0–2 (Figure 8).

### Human Population Density

Human population density was significantly higher in buffer areas around historical than around recent records (Table 3): 66% of buffer areas around historical records were allocated to the high human population density classes 0–2, whereas only 30% of buffer areas of recent records belonged to these classes.

### Protected Areas and Prediction of Key Localities

**Figure 9:**
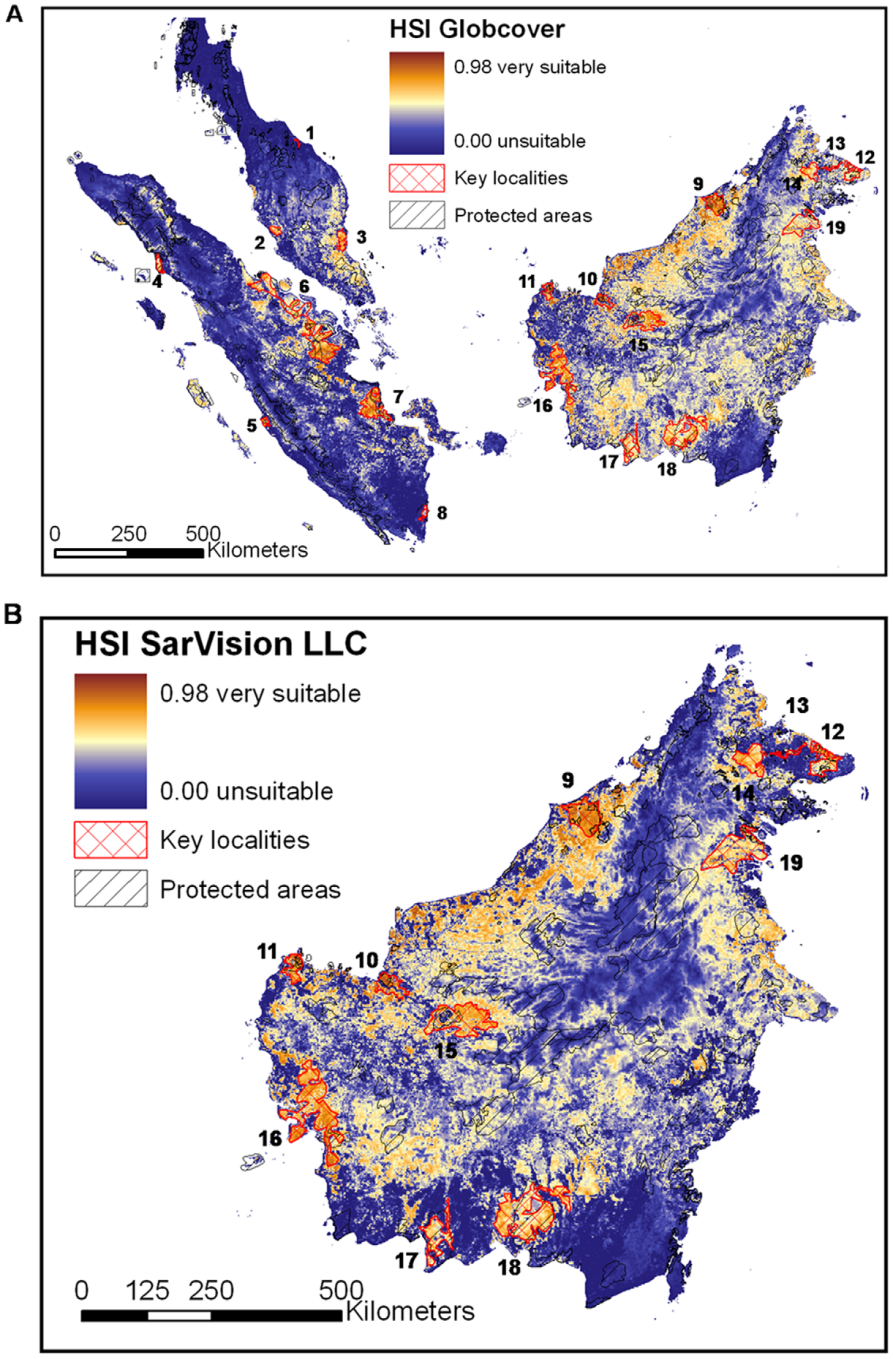
Habitat suitability map (based on the habitat suitability index HSI) for GlobCover data and SarVision LLC. For comparison, currently protected areas and predicted key localities 1–19 (Table 6) identified in this study are illustrated.

**Figure 10:**
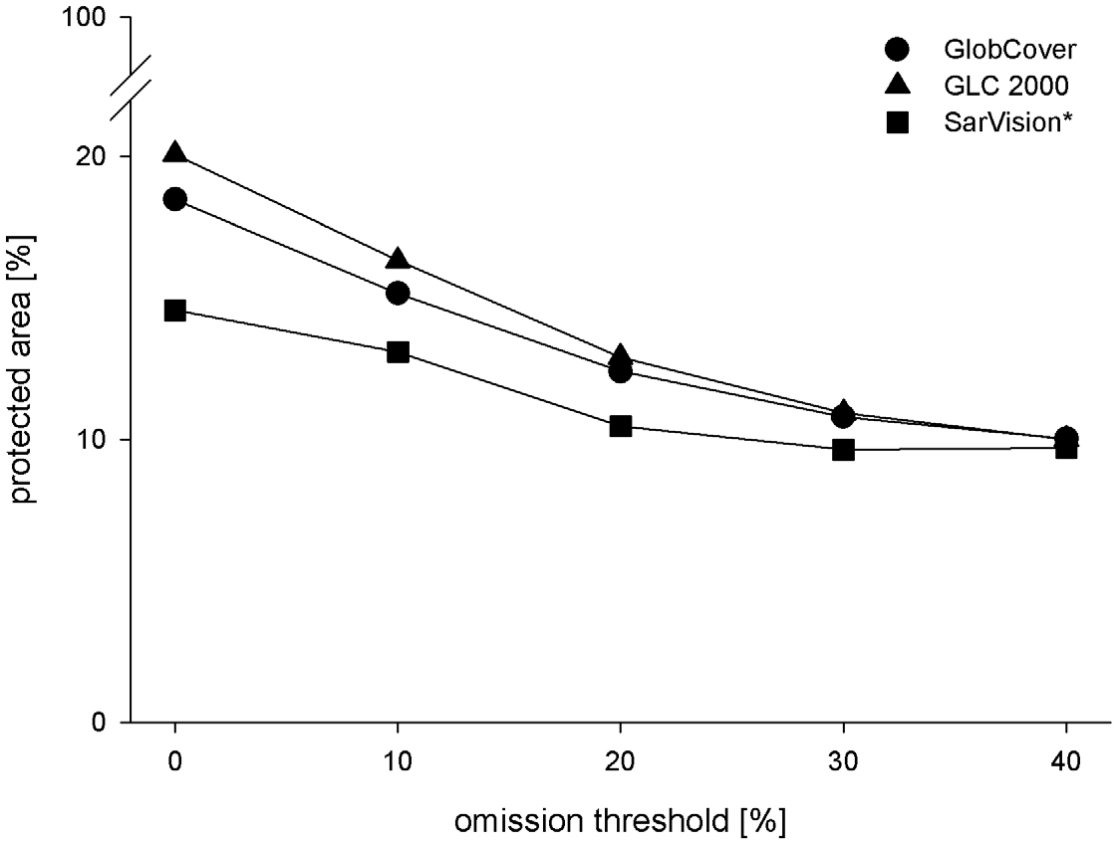
Relative proportions of predicted habitat under protection according to the World Database of Protected Areas under the five omission threshold scenarios.

Figure 9 shows the probability of occurrence of flat-headed cats based on the habitat suitability index HSI together with the location of protected areas. The proportion of suitable habitat under total protection in the three land cover maps and five omission scenarios indicated that currently between 10% and 20% of suitable habitat is under total protection as defined by the IUCN [42]. The proportion under total protection decreased with the higher omission thresholds in all three land cover maps (Figure 10). The proportion of protection of all forested areas was 22% for GlobCover, 23% for SarVision LLC and 24% for GLC 2000 classification. Based on Figure 9 we identified 19 forest areas throughout the entire range of the flat-headed cat which are likely to make significant contributions to the long-term survival of the flat-headed cat (Table 6).

**Table 6:** Predicted key localities for the conservation of the flat-headed cat.

No*	Name of Forest		Remarks
**Peninsular Thailand/Malaysia**			
1	Toh Daeng Peat Swamp forest (Thailand)		isolated, small
2	Selangor Peat Swamp Forest (Malaysia)		partly degraded
3	Pahang Peat Swamp Forest (Malaysia)		fragmented
**Sumatra**			
4	SW part of Gunung Leuser NP & Singkil Barat Nature Reserve		good
5	Hutan Lunang Nature Reserve		small
6	Kerumutan Wildlife Reserve & forest to the E & N, including Belat Besar Linau up to Senepis Buluhala		large, but fragmented
7	Berbak National Park & adjacent inland forests along the Merang River		good
8	Way Kambas National Park		small
**Borneo**			
9	East Brunei, including Tasek Merimbun and forests to the SE, Belait Peat Swamp, Ulu Badas, Bukit Sawat, Ulu Mendaram (Brunei Darussalam)		very good
10	Maludam NP (Sarawak, Malaysia)		good, partly fragmented
11	Samusam Wildlife Sanctuary & adjacent forests to the W, Hutan Sambas Nature Reserve & adjacent forests to the S (Sarawak, Malaysia)		comparatively small
12	Tabin Wildlife Reserve & Kulamba Wildlife Reserve & adjacent coastline peat swamp forests (Sabah, Malaysia)		good
13	Kinabatangan Wildlife Sanctuary (Sabah, Malaysia)		small, highly fragmented
14	Deramakot/Tangkulap/Segaliud Lokan/North Malua commercial forest reserves (Sabah, Malaysia)		good, commercially used
15	West Kalimantan, Danau Sentarum & forests to the W		large
16	West Kalimantan, S of Pontianak along the coast to Gunung Palung NP, including Pulau Maya (Kalimantan, Indonesia)		good, but mostly unprotected
17	Tanjung Puting (Kalimantan, Indonesia)		large, but isolated
18	Sabangau Peat Swamp Forest & Adjacent areas W & NE of the protected area (Kalimantan, Indonesia)		large and mostly contiguous, only partly protected
19	East Kalimantan, Muara Sebuku Nature Reserve & large area south of this reserve (Kalimantan, Indonesia)		good, but mostly unprotected

*refers to numbers shown on maps in Figure 8a and 8b; SW  =  southwest; E  =  east; N  =  north, SE  =  southeast, W  =  west, S  =  south, NE  =  northeast; NP  =  National Park.

## Discussion

### Location of Historical and Recent Records

Our database of all recent and historical records of the flat-headed cat (Table S1) is provided to assist further conservation plans and will serve as a useful starting point for the collation of future sightings of this endangered species. Identifying the precise location for most records was not easy but as long as errors in the assignment of coordinates are random with respect to the habitat composition of the calculated buffer areas, this will increase the overall error but not bias the results and is unlikely to affect the principal conclusions.

The number of sightings and camera-trapping pictures with precise location information was low, even though the number of camera-trapping studies in South-East Asia has increased during the past 20 years and we had first-hand information from many of these studies. Information from 17 camera-trapping photographs was available for use in this study. This is the lowest number of photographs from camera-traps of any South-East Asian felid and is a testimony to the extremely limited knowledge regarding this species and possibly the limits of its range and distribution. For instance, in Sumatra the presence of tigers (*Panthera tigris*), Asian golden cat (*Pardofelis temminckii*), marbled cat (*Pardofelis marmorata*), Sunda clouded leopard (*Neofelis diardi*) and leopard cat is regularly confirmed by camera-trap photographs. On Borneo, an increasing number of camera-trapping studies documented the regular occurrence of the leopard cat, Sunda clouded leopard, marbled cat and the Borneo endemic bay cat (*Pardofelis badia*). Even though records of the latter species were very rare [43], its geographic range and distribution might now be better understood than that of the flat-headed cat [44], [45].

As far as we are aware there are no camera-trap records of the flat-headed cat from Thailand and Peninsular Malaysia. However, most camera-trapping initiatives are designed to record large cat species, typically tigers in Peninsular Thailand/Malaysia and on Sumatra. For this purpose, cameras are placed along roads or ridges, habitats unlikely to be used by flat-headed cats and therefore inappropriately to record this species. Almost none of the camera-trapping studies placed their cameras along the edges of lakes, ponds or rivers. This could easily lead to a sampling bias and thus may affect capture probabilities of flat-headed cats.

### Habitat Selection

The GIS analysis is a first step towards identifying habitat preferences of the flat-headed cat. Our results indicate that the most relevant factor explaining its distribution is the distance to freshwater sources such as major rivers and large lakes. Although with the currently available DCW map it was not possible to assess distances to the nearest minor freshwater source, our results suggest that larger watercourses and water bodies are needed and that small ones alone are unlikely to be sufficient, maybe because they face a higher risk to dry up.

This assumption is supported by the most important bioclimatic variable, precipitation during the driest month, which suggests that rainfall during the dry season is a critical factor for the presence of flat-headed cats, presumably because the rainfall supports the persistence of small-sized water bodies. This might also explain why the range of the flat-headed cat does not expand further north, as the climate is more seasonal there [46]. The model also predicted that South Kalimantan (Figure 3) provides no suitable habitat for the flat-headed cat. This may also be explained by climatic conditions, as this region has typically a more severe dry season [47].

Most flat-headed cat records were located in extreme lowland areas below 100 m asl. The difference in altitude between historical and recent records might be a consequence of sampling bias in historical times. For logistical reasons, collectors of museum specimens many decades ago spent disproportionately more time in lowland areas along coastlines or rivers. Even though roads or helicopters provide access to more remote areas at higher altitudes today, most recent records are still from the lowlands at less than 100 m asl.

### Range and Distribution

For species with limited information about their historical and current range, predictive models are a useful tool to assist conservation planning [19], [21], [48]–[49]. The flat-headed cat is one of the least known felids worldwide and its special habitat requirements make it an ideal candidate for the application of predictive modelling. The predicted potential former distribution (Figure 3) and the map of current habitat suitability (Figure 9) differ substantially from the recently published distribution map in the most recent status assessment for the IUCN Red List (Figure 2) [3]. Our maps suggest that coastal and lowland areas are key habitats for the flat-headed cat, but these are mostly missing from the IUCN distribution map. Although the Red List was reassessed in 2008 and the increasing threat to the flat-headed cat was recognised by the species assessors (all authors of this paper: AH, JS, JR, AW & SS), the distribution map of the 1996 Cat Action Plan map [5] was only slightly modified by the IUCN with the help of the GLC 2000 land cover classification. This must have led to an overemphasis on large forest tracts at higher altitudes and a neglect of smaller, already more fragmented and anthropogenically modified coastal forests.

### Loss of Suitable Habitat and Human Population Growth

This study provides a first indication of how much of flat-headed cat habitat has already been converted into unsuitable habitat. South-East Asia has one of the world's highest deforestation rates with an annual forest reduction of 1.3% [50] and by 2100 three quarters of its original forests are expected to be lost [51]; on Borneo alone 1.3 million ha of lowland forest are lost annually [52]. The high levels of habitat reduction (between 54% and 68%) as estimated by our models indicate that the actual loss of habitat is immense. This may even be a conservative estimate of loss, since our model accepted remaining forest patches of any size as suitable habitat even though it is likely that small and fragmented forests will not support viable populations of flat-headed cats.

A second estimate of loss of suitable habitat was obtained from the land cover in buffer areas around historical locations. Between 54% (GlobCover) and 80% (SarVision) of previously suitable habitat has already been transformed to unsuitable or poor habitats in the land cover categories 0 to 2 (Figure 8). That between 31% and 42% of the habitat in buffer areas around recent records belongs to categories of unsuitable habitat reflects that some of our “recent” records were collected 25 years ago. Given current deforestation rates much suitable habitat has vanished in the interim. Two records from Sumatra provide good examples for this land cover and land use change. One record from the Riau Province involved a flat-headed cat trapped by hunters in a small forest patch between oil palm plantations, and a second record from Tapan came from an area which today is already transformed into oil palm plantations.

Similarly, the data on human population density showed that 66% of historical records are at present surrounded by high human population densities (Figure 8), resulting from the rapid population growth rates in South-East Asia [53].

### Other Threats to the Flat-Headed Cat

Our analysis considered habitat loss and human population density, but not proximate threats to flat-headed cats such as hunting, over-fishing or fresh-water pollution. Presumably environmental pollution, due to gold mining and agricultural practices is largely reducing distribution and long-term survival prospects of this species. Malaysia and Indonesia are already the largest producers of palm oil [54]–[56] and the globally increasing demand for biofuel and other products derived from palm oil further threatens the remaining forested areas [54]. Expansion of plantations often involves wetland drainage and loss of upstream forests. This results in reduced water runoff during the drier months and may therefore, given the relationship of flat-headed cat distribution to dry season rainfall levels, contribute to the loss of habitat. As we could not incorporate these threats into our estimates, the observed loss of suitable habitat is likely to be conservative. These threats require urgent and careful further elucidation, since the long-term consequences of large-scale agrochemical use, especially in oil palm plantations, are uncertain.

### Protected Areas

Our results show that currently only between 10% and 20% (where 20% refers to the 0% omission scenario) of potentially suitable habitat is classified as completely protected by IUCN criteria. For the other scenarios the area under full protection was calculated in the range between 10% and 16%. This is a disproportionally low percentage since–throughout the study region - between 22% and 24% (depending on the land cover classification) of the forested area is fully protected. The discrepancy is explained by large national parks, such as Taman Negara, Kerinci, Gunung Leuser or Sungai Kayan and Sungai Mentarang, which are located in more mountainous regions and do not contain large areas of suitable habitats for the flat-headed cat. Owing to the low proportion of totally protected flat-headed cat habitats it can be assumed that currently suitable areas will be converted into plantations in the near future. Higher omission rates in the model reduce the fraction of potential habitats giving a focus on core distribution areas. If protected areas were located within core zones of the distribution of the flat-headed cat, the proportion of protected areas should increase with higher omission rates. Our data show the opposite effect (Figure 10), indicating that protected areas are mainly located within marginal habitats and areas with a lower probability of flat-headed cat occurrence. This highly alarming result is not surprising since well-watered lowlands are among the areas most desired for expansion of agriculture and industry, leading to a general disinclination to declare large protected areas within them. The World Database of Protected Areas only includes those that meet IUCN criteria. Although some of the protected areas not included in this database do contain the flat-headed cat (e.g., the commercially used forests in and around Deramakot Forest Reserve in Sabah), without any doubt the proportion of the flat-headed cat distribution range under any level of official protection is very low.

### Key Localities in Peninsular Thailand and Malaysia

Peninsular Thailand/Malaysia has the worst perspective for the long-term survival of the flat-headed cat. No single continuous forest block comprises potential flat-headed cat habitat: all the larger forest reserves and national parks in Peninsular Thailand/Malaysia are located in the interior of the country, away from the species' predicted distribution range. In southern Thailand, Toh Daeng Peat Swamp forest and a few very small forest patches might be the only areas where flat-headed cats still occur in Thailand. In Peninsular Malaysia, we identified only two suitable forest areas, the Selangor peat swamp forest (west coast) and the Pahang Peat Swamp Forest (east coast). Recent sightings from Pahang Peat Swamp Forest raise hope that these forests still support a population of flat-headed cats. However, even this forest patch is already threatened and degraded into several smaller forest blocks.

### Key Localities in Sumatra

Sumatra holds five key areas for the flat-headed cat. Two of these, Way Kambas National Park and Hutan Lunang Nature Reserve, are already small and isolated. Camera-trapping pictures from Way Kambas suggest that this wildlife reserve might have the potential to protect the flat-headed cat at the most southern part of its distribution range [57]. In northern Sumatra, south-western Gunung Leuser National Park, close to the coastline, with the adjacent Singkil Barat Nature Reserve comprises an area of great potential for the flat-headed cat. A recent camera-trapping picture from Suak (G. M. Fredriksson *in litt*.) supports this conclusion from the model. Recent sightings along Merang river in South Sumatra and from Berbak National Park [6], [58] endorse our prediction that this area is important for the flat-headed cat. The largest suitable Sumatran forest for the flat-headed cat is in Riau Province. This large forest block ranges from Senepis Buluhala in the north, where the Sumatran Tiger Conservation Project obtained a camera-trap photograph, to the forests of Kerumutan Wildlife Reserve in the south. It is already fragmented and Riau has the highest deforestation rate in Indonesia, having lost 65% of its forest in the last 25 years [59]. Within this area there is a strong move by several NGOs to protect the remaining forest specifically for Sumatran tigers, but these efforts also need to include the lowland swamp forest in order to protect species depending on wetlands such as the flat-headed cat.

### Key Localities in Borneo

Borneo covers the largest part of the range of the flat-headed cat and holds most of the potentially suitable habitats. Based on our model predictions using topo-climatic conditions, the Malaysian state of Sarawak historically had the largest suitable flat-headed cat areas. However, the lowland coastal areas have borne the brunt of recent land development and agricultural expansion, so that today most natural forests are found in the interior, the least favourable habitats for the flat-headed cat as predicted by our model. We identified two areas in Sarawak which most likely hold viable flat-headed cat populations: the comparatively smaller Samusam National Park and the larger Maludam National Park. Both are isolated areas located along the north coast of Borneo. In addition to these two key localities recent sightings from Loagan Bunut National Park reveal the importance of this small national park for the flat-headed cat in Sarawak.

Sabah holds three lowland forest complexes. The largest is Tabin Wildlife Reserve (where Yasuda *et al*. obtained camera-trap photographs [60]) together with the northern coastal areas and Kulamba Wildlife Reserve. Currently, peat swamp and mangrove forests in the north are not completely protected, but if conserved they could potentially link this forest block with the Kinabatangan Wildlife Sanctuary (WS), a series of small forest patches along the lower Kinabatangan River. Although the Kinabatangan WS is highly fragmented, the high number of flat-headed cat sightings (mainly by tourists on night river cruises) along the tributaries of the Kinabatangan shows that this wildlife sanctuary is still a suitable area for the flat-headed cat (Video S1). The third forest complex identified in Sabah is an area south (Northern Malua) and north (Deramakot, Tangkulap & Segaliud Lokan) of the upper Kinabatangan. All forest reserves located in this forest complex are for commercial timber production under low-impact selective logging strategies, including a long felling cycle and extended regeneration times, and the pursuit to be certified by the Forest Stewardship Council (Deramakot and Tangkulap) or the Malaysian Timber Certification Council (Segaliud Lokan). The high number (15) of recent camera-trap photographs from this forest block [61] (AW & AM unpublished data) indicates that low-impact commercial use may not be entirely incompatible with the habitat requirements of flat-headed cats. In addition to these three key habitats in Sabah, flat-headed cats were recently reported in the Ulu Segama FR (AJH & JR) and in Maliau Basin (CT) but as these areas are mainly surrounded by upland forests we did not include them in the list of key localities.

In Brunei Darussalam, the large undisturbed forests of East Brunei with the completely protected areas of Tasek Merimbun (where Yasuda *et al*. obtained camera-trap photographs [60]), Ulu Mendaram and Belait peat swamps are the main shelter for flat-headed cats. This area is one of the largest undisturbed forest complexes in Borneo.

As in the other parts of the study region, most undisturbed and protected areas in Kalimantan are in the inland uplands. However, several larger forest blocks remain along the coastline and in lowland areas. In West Kalimantan the Danau Sentarum forest complex, one of the few large inland forest blocks, was predicted by our model to contain appropriate habitats for the flat-headed cat. A second key locality in West Kalimantan, which is currently mainly unprotected, ranges from south of Pontianak along the coastline to Gunung Palung National Park which was predicted by our model to be a good flat-headed cat habitat. In the southern part of Central Kalimantan we identified two interesting forest blocks. Tanjung Puting along the south coast is a large, already relatively isolated area. With an area of 5,300 km^2^, the Sabangau Peat Swamp Forest is the largest remaining peat swamp forest in Indonesia [62]. In the vicinity of this protected area, large forests suitable for the flat-headed cat as predicted by our model remain. Its enormous size and the recent evidence of flat-headed cats from this area [63] identify this forest as one of the most important sites for the flat-headed cat. However, this area is threatened by fires and drainage, as are many of the peat swamp forests in Kalimantan. In East Kalimantan, the Muara Sebuku Nature Reserve and the larger forests to its south might have the potential to be a home for flat-headed cats. In Kalimantan, recent records also come from more upland forests [64] (Wulfraat *in litt*.). Although these sightings may suggest that flat-headed cats follow rivers upstream, we did not include these areas in our selection of key localities as there are only very few records obtained so far.

### Conclusion

This overview of the potential former and recent distribution of the flat-headed cat is a first step to improve conservation efforts for this threatened felid. Verification of model predictions by field surveys is urgently needed, with camera-trapping efforts directed towards: (1) areas around lowland freshwater sources within forests where flat-headed cats are more likely to be detected; (2) plantation-dominated landscapes to corroborate–or cast doubt on–Khan's records of them living in plantations [12] for which we could not find any support and where further research needs to show whether flat-headed cats occur at forest edges and enter plantations; (3) small forest fragments that have been isolated for decades to see what sort of minimum patch size is realistic; (4) upland forests along major rivers and on well-watered upland plateaux with good forest cover to see how much of a role, if any, these areas could play in the species conservation; (5) investigations of large landscapes of logging estates to see if the findings from Deramakot [61] are generally representative. Postulations that flat-headed cats and other swamp forest specialists such as the otter civet (*Cynogale bennettii*) or the hairy-nosed otter (*Lutra sumatrana*) live in low-impact logging landscapes would give a high support to argue for their maintenance as low-impact logging estates rather than converting them into plantations.

Besides the intensification of large-scale survey efforts in lowland, peat swamp and mangrove forests, detailed site-specific multiple year studies using different methods simultaneously (e.g. radio-tracking, camera-trapping) would be needed to refine current knowledge on habitat and food preferences and requirements. Suitable areas for this purpose are the Pahang Peat Swamp Forest, Senepis Buluhala, Sabangau Peat Swamp Forest, Tasek Merimbun National Park, Kinabatangan Wildlife Sanctuary or Deramakot Forest Reserve where flat-headed cats have been recorded recently.

Our study showed that large areas of the predicted former distribution range have already been transformed into croplands or plantations, and based on the records from this study and our experiences we cast doubt on the statement that flat-headed cats live and reproduce in plantations. Furthermore, the low proportion of key forest areas under complete protection emphasises the urgency of further conservation action. We consider the flat-headed cat suitable to serve as a flagship species for the protection of several other endangered species associated with the threatened tropical lowland and peat swamp forests.

## Supporting Information

Table S1 Summary of recent and historical records of the flat-headed cat *Prionailurus planiceps*.
(0.04 MB XLS)

Video S1 Flat-headed cat from the Kinabatangan Wildlife Sanctuary (ConCaSa).
(7.56 MB MP4)
